# The AFU Campus Inventory Tool for Assessing Age Friendliness in the Objective Environment

**DOI:** 10.1093/geroni/igaa057.1739

**Published:** 2020-12-16

**Authors:** Susan Whitbourne, Nina Silverstein, Lauren Bowen, Taylor Jansen

**Affiliations:** 1 University of Massachusetts Amherst, Boston, Massachusetts, United States; 2 University of Massachusetts Boston, Needham, Massachusetts, United States; 3 University of Massachusetts, Boston, Boston, Massachusetts, United States; 4 University of Massachusetts Boston, Boston, Massachusetts, United States

## Abstract

The AFU principles establish a framework that universities can use to set aspirational goals. As AFU principles become more widely adopted, there will be a need for institutions of higher education to evaluate their progress toward achieving these goals. In part, the AFU goals represent structural elements of the campus, or the “objective” environment. The AFU Campus Inventory provides an efficient, form-based, reporting tool for use by relevant campus departments to provide information on age-friendly practices, such as the prevalence of older people on campus as faculty, staff, and students; the provision of courses on aging, advising for older community members, and such amenities as reduced prices for various services. In this session, the Inventory will be demonstrated utilizing preliminary data from campuses in Massachusetts to illustrate its feasibility. Participants will be shown how to use the Inventory for adaptation at their own institutions.

